# Heart rate variability in sciatica patients referred to spine surgery: a case control study

**DOI:** 10.1186/1471-2474-14-149

**Published:** 2013-04-26

**Authors:** Jarmo Södervall, Jaro Karppinen, Jukka Puolitaival, Eero Kyllönen, Antti M Kiviniemi, Mikko P Tulppo, Arto J Hautala

**Affiliations:** 1 Department of Exercise and Medical Physiology, Verve Research, Kasarmintie 13, P.O. Box 404, Oulu, 90101, Finland; 2 Department of Physical and Rehabilitation Medicine, Institute of Clinical Medicine, University of Oulu, Oulu, Finland; 3 Finnish Institute of Occupational Health, Work and Health Ability, and Disability Prevention Centre, Oulu, Finland

**Keywords:** Autonomic nervous system, Back pain, Sciatica, Sympathovagal balance, Vagal activity

## Abstract

**Background:**

A chronic pain condition may result in altered autonomic nervous system regulation in various patient populations. We evaluated whether autonomic regulation differs between sciatica patients referred to spine surgery and age-matched healthy controls analyzed with heart rate variability techniques (HRV).

**Methods:**

HRV of patients (n = 201) and healthy controls (n = 138) were measured in standing conditions (5 min). High frequency (HF) power as an index of cardiac vagal modulation and the low-to-high-frequency (LF/HF) ratio and short-term fractal scaling exponent α_1_ as indices of sympathovagal balance were analyzed. Pain intensity was assessed on a Visual Analog Scale (VAS) and perceived disability with Oswestry Disability Index.

**Results:**

The Oswestry and VAS scores were higher in the patients than in the controls (p < 0.0001 for both). HF power was markedly lower for the patients compared to the controls (p < 0.0001). The LF/HF ratio and α_1_ were higher in the patients than in the controls (p < 0.01 for both). After adjusting for sex, smoking, BMI, and leisure-time physical activity, HF power (p = 0.011) and α_1_ (p = 0.012) still differed between the groups. Among the patients, HF power was slightly associated with the duration of chronic pain (r = −.232, p = 0.003).

**Conclusions:**

Sciatica patients referred to spine surgery had altered cardiac autonomic regulation expressed as decreased vagal activity and an increased sympathovagal balance toward sympathetic dominance when compared with age-matched healthy controls.

## Background

Low back disorders, including low back pain (LBP) and sciatica, are common health problems among adults and represent a significant burden on health care systems globally. A population-based survey showed that 19% of the subjects had chronic pain, and LBP was the main reason in almost half of these patients [1]. Sciatic pain is more severe and the outcome of this pain is worse than localized LBP [2]. Furthermore, it causes an increased risk of hospitalization [3], longer absences due to illness, and more disability than does nonspecific LBP [4,5].

Various states of chronic pain are associated with altered autonomic nervous system function, assessed with the heart rate variability (HRV) indices, e.g. patients with fibromyalgia have shown lower HRV [6] and blunted autonomic response to an orthostatic challenge [7]. Decreased HRV has also been reported in patients with complex regional pain syndrome [8] and in chronic neck-shoulder pain patients [9]. Likewise, signs of altered autonomic regulation in subjects with chronic LBP have been observed. An earlier study on chronic LBP showed that HRV was lower among patients with a moderate perceived disability (Oswestry 20–40%) than among those with a minimal disability (Oswestry <20%). Interestingly, a significant association was found between HRV and perceived physical impairment, but not between HRV and subjective pain evaluated on a numerical rating scale [10].

Depressed HRV has been shown to be associated with an adverse prognosis in general populations as well as in different patient groups [11-16]. The function of the autonomic nervous system in sciatica patients referred to spine surgery is not well understood and, to our knowledge, has not been meticulously studied in relation to various spectral and non-linear dynamics of HRV. Therefore, we examined whether HRV differs between sciatica patients referred to spine surgery and age-matched healthy controls. We hypothesized that HRV is altered in sciatica patients compared with healthy controls, and secondly, that HRV is associated with pain intensity and perceived disability in sciatica patients.

## Methods

### Study population

The study population consisted of voluntary consecutive patients (n = 201, age 42 ± 11 years, 113 men and 88 women) referred to evaluation of spine surgery because of radicular symptoms in the Departments of Orthopedic Surgery and Neurosurgery at the Oulu University Hospital between June 2007 and January 2011. A neurosurgeon or orthopedic surgeon investigated the patients and a lumbar magnetic resonance imaging (MRI) was performed to all. Altogether 88% (n = 176) of patients had MRI-verified disc herniation (HNP)-induced nerve root impingement and major part of these patients were operated consequently. Twelve percent (n = 25) of patients did not have HNP-induced sciatica; about one half of them had radicular pain but in MRI only disc degeneration with or without bulge at the suspected level, while the rest had spondylolisthesis, nerve root canal narrowing or ligamentum flavum thickening. Patients with symptomatic spinal stenosis or spondylolisthesis on basis of the referral were excluded. The control subjects (n = 138, age 42 ± 11 years, 39 men and 99 women) were healthy volunteers without low back pain, back disease, or back surgery and they were recruited to the study by the local occupational health care center (ODL Health, Oulu, Finland). All the subjects were asked not to eat or to drink coffee for 3 h before the tests. In addition, strenuous physical activity and alcohol consumption were prohibited on the day of the test and the preceding day. MRI of the lumbar spine was performed for both groups with 1.5-T equipment (Signa, General Electric, Milwaukee, WI, and Magnetom Avanto, Siemens, Erlangen, Germany). A routine spine MRI protocol was used, including sagittal T1- and T2-weighted images of the entire lumbar spine. The subjects’ place of residence was mainly the city of Oulu (population ca. 140,000) or the neighboring municipalities, but the enrollment area was comprised of the two northernmost provinces of Finland (Oulu and Lapland). All the subjects filled out a baseline questionnaire. Potential confounders or modifying factors including comorbidities, leisure-time physical activity (LTPA), and smoking status were collected with the questionnaire. The demographics and clinical characteristics of the study cohort are presented in Table 1. The Ethical Committee of the Northern Ostrobothnia Hospital District, Oulu, Finland, approved the protocol, and all the patients gave informed consent.

**Table 1:** Demographic and clinical characteristics in patients referred to spine surgery and controls

**Variable**	**Patients (n = 201)**	**Controls (n = 138)**	**P-value**
Men/Women	113 (56) / 88 (44)	39 (28) / 99 (73)	<0.0001
Age, yr	42 ± 11	42 ± 11	0.929
BMI, kg/m^2^	26.4 ± 4.4	23.9 ± 3.6	<0.0001
Smoking	124 (62)	49 (36)	<0.0001
LTPA			
High	52 (28)	55 (40)	0.014
Moderate	111 (59)	67 (49)	0.091
Low	26 (13)	14 (11)	0.349
Oswestry, %	37 ± 14	2 ± 3	<0.0001
VAS, cm	5.8 ± 2.5	0.6 ± 1.0	<0.0001
Pain Duration, days	193 ± 119	7 ± 16	<0.0001
Hypertension	20 (10)	7 (5)	0.075
Diabetes	3 (1)	1 (<1)	0.462
CAD	3 (1)	2 (1)	0.671
Hypercholesterolemia	6 (3)	3 (2)	0.464
Asthma	7 (3)	7 (5)	0.324
Arthritis or Fibromyalgia	4 (2)	1 (<1)	0.329

Values are means ± SD or number of subjects (%).

BMI = body mass index, LTPA = leisure-time physical activity, Oswestry = low back pain disability index, VAS = Visual Analog Scale, CAD = coronary artery disease.

### Assessment of pain and disability

All the subjects reported the duration of LBP during the past year. The diagnoses for surgical referral were obtained from hospital records. Back-related disability was assessed using the Oswestry Disability Index [17]. A 10-cm Visual Analog Scale (VAS) was used to define the intensity of LBP [18], with ‘0’ representing a lack of pain and ‘10’ the worst imaginable LBP.

### Assessment of heart rate variability

A heart rate (HR) monitor (Polar RS800, Polar Electro Oy, Kempele, Finland) was used to record R-R intervals with a timing accuracy of 1 ms during 5 minutes (min) in a supine position followed by 5 min of standing in a quiet room after 5 min of rest in a supine position. The recordings were saved on a computer for further analysis of HRV with HEARTS software (Heart Signal Co, Kempele, Finland). To exclude all undesirable beats, which accounted for <1% of the data for each subject, the R-R intervals were edited by visual inspection. HRV indices were calculated separately for the 5-min supine and standing conditions.

Mean HR and the standard deviation of normal-to-normal R-R intervals (SDNN) were used as time-domain measures of HRV. An autoregressive model (model 18) was used to estimate the power spectrum densities of R-R interval variability. Vagally mediated HF power (0.15 to 0.4 Hz), both sympathetically and vagally mediated low frequency power (LF, 0.04 to 0.15 Hz), and the low-to-high-frequency ratio (LF/HF ratio) as an index of sympathovagal balance were calculated [19]. A Poincaré plot was analyzed quantitatively using two-dimensional vector analysis. Briefly, SD1 measures the magnitude of vagally mediated beat-to-beat variability in the data. Details of the two-dimensional vector analyses have been described previously elsewhere and were used accordingly in the present study [20]. As non-linear indices of HRV we analyzed the short-term (α_1_) fractal scaling exponent. The fractal analysis method differs from traditional measures of HRV because it does not measure the magnitude of variability, but rather the qualitative characteristics and correlation features of HR behavior. Briefly, in the fractal analysis, the root-mean-square fluctuations of integrated and detrended data are measured in observation windows of different sizes and then plotted against the size of the window on a log-log scale. In this study, short-term (from 4 to 11 beats) α_1_ was calculated on the basis of previous experiments [21,22]. Details of the fractal scaling method have been described previously elsewhere [23].

### Statistical analyses

The normal Gaussian distribution of the data was verified with the Kolmogorov-Smirnov goodness-of-fit test. Because the spectral components of HRV had a non-Gaussian distribution, a natural logarithmic transformation was performed on both. Analysis of variance (ANOVA) was used to compare if the demographics and clinical characteristics and the mean values of HRV differed between the patients and the controls. A chi-square test was also used to compare categorical parameters. Analysis of covariance (ANCOVA) with 95% confidence intervals (CI) was performed to compare the differences in HRV between the groups, with gender, body mass index (BMI), smoking status, and high LTPA used as covariates. Pearson correlation analysis and ANOVA were applied to study the associations between HRV and demographics, disability, and pain-related parameters among the patients. Since underlying co-morbidities may be associated with altered HRV, we excluded patients with hypertension, diabetes, coronary artery disease, hypercholesterolemia, asthma and arthritis or fibromyalgia from both patient and control populations and reperformed the statistical analyses. Furthermore, we performed separately a sub-analysis by comparing patients with and without HNP-induced sciatica. The SPSS statistical software package (IBM SPSS Statistics 20 for Windows, SPSS Inc., USA) was used for the analyses. A P-value <0.05 was considered statistically significant.

## Results

### Study population characteristics

The patients and controls did not differ with respect to age, co-morbidities, or moderate or low LTPA (Table 1). However, BMI was 10% higher in the patients than in the controls (p < 0.0001) and there were more smokers among the patients than among the controls (62% vs. 36%, respectively, p < 0.0001). There were more men (56%) in the patient group compared with the controls (28%) (p < 0.0001). High LTPA was reported among 40% of the control subjects, whereas 28% of the patients showed high LTPA (p < 0.014).

### Heart rate variability

**Figure 1:**
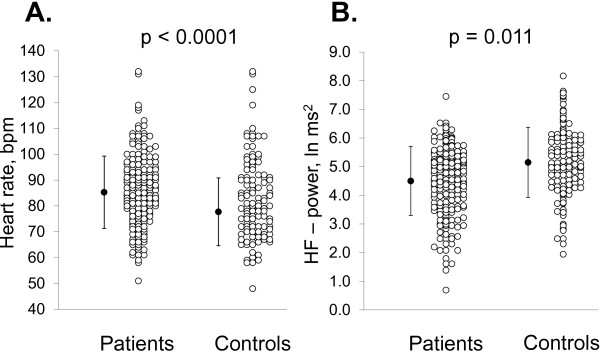
Mean heart rate (A) and vagally mediated HF power (B) in a standing position for 5 minutes for patients and controls.

The differences in HRV between the patients referred to spine surgery and the controls are presented in Table 2. HR was 4 beats higher among the patients in a supine position compared with the controls (p = 0.002) and 7 beats higher in a standing position (p < 0.0001, Figure 1A). The difference in all HRV parameters between the patients and the controls (p = 0.002 to p < 0.0001) in a standing position was statistically significant, e.g. vagally mediated HF power (Figure 1B) was 13% lower and the LF/HF ratio, as an index of sympathovagal balance, was 36% higher in the patients than in the controls. After adjustment for gender, BMI, smoking, and high LTPA, the statistical difference between the groups in a standing position remained evident for HR, HF power, SD1, and α_1_ (Table 2). When underlying co-morbidities were excluded from the analyses, the results remained the same; e.g. HF power, SD1, and α_1_ in standing position differed significantly between the groups after adjustments (p = 0.048, p = 0.012 and p = 0.010, respectively). Furthermore, when comparing patients with and without HNP-induced sciatica, patients without HNP-induced sciatica were older (50 ± 7 vs. 40 ± 11, p < 0.0001) and the duration of pain was longer (266 ± 139 vs. 181 ± 107 days, p = 0.002) compared to patients with HNP-induced sciatica. The two patient groups differed also in HRV; e.g. HF power in standing position was 5.63 ± 1.36 in HNP-induced sciatica patients and 4.99 ± 1.36 (p = 0.002) in patients without HNP-induced sciatica. However, after adjustment with age and pain duration the difference disappeared in all HRV parameters (e.g. in HF power in standing position p = 0.211).

**Table 2:** Heart rate variability indices in patients referred to spine surgery and controls during 5 minutes of supine and standing conditions

	**Supine**				**Standing**			
	**Patients**	**Controls**	**Mean difference (95% CI for difference)**	**Adj. P-value**	**Patients**	**Controls**	**Mean difference (95% CI for difference)**	**Adj. P-value**
HR, bpm	69 ± 13	65 ± 10	3 (0 – 6 )	0.051	85 ± 14	78 ± 13	7 (3 – 10)	<0.0001
SDNN, ms	45 ± 28	48 ± 24	2 (−5 – 9)	0.598	51 ± 22	61 ± 27	−6 (−12 – 0)	0.065
HF power, ln ms^2^	5.6 ± 1.4	6.0 ± 1.3	−0.2 (−0.5 – 0.2)	0.356	4.5 ± 1.2	5.1 ± 1.2	−0.4 (−0.7 – -0.1)	0.011
LF power, ln ms^2^	6.0 ± 1.1	6.1 ± 1.0	−0.1 (−0.4 – 0.2)	0.404	6.3 ± 1.1	6.6 ± 0.9	−0.2 (−0.5 – 0.1)	0.074
LF-to-HF ratio	2.0 ± 1.7	1.9 ± 2.3	−0.3 (−0.8 – 0.2)	0.205	7.9 ± 6.4	5.8 ± 5.2	1.0 (−0.4 – 2.5)	0.159
SD1, ms	23 ± 15	28 ± 21	−2 (−6 – 3)	0.443	13 ± 7	18 ± 12	−4 (−6 – -1)	0.002
α_1_	1.09 ± 0.23	1.01 ± 0.29	0.03 (−0.03 – 0.10)	0.304	1.58 ± 0.22	1.49 ± 0.24	0.73 (0.02 – 0.13)	0.012

Values are means ± SD. Mean differences and 95% confidence intervals (CI) for differences between groups are given after adjustment for gender, BMI, smoking status and high LTPA.

HR = heart rate, SDNN = standard deviation of all R-R intervals, HF = high frequency, LF = low frequency, SD1 = beat to beat R-R interval fluctuation, α_1_ = short-term fractal scaling exponent.

### Relationships between heart rate variability and other variables in the patients

The Oswestry and VAS scores did not correlate with HRV, e.g. the correlation values between HF power and the Oswestry score and HF power and the VAS score in a standing position were −0.036 and −0.094, respectively. Pain duration was inversely associated with standing HF power (r = −.232, p = 0.003) but not with HR, SD1, or α_1_ (r = −0.032, -0.111, 0.001, respectively). Similar results were found when underlying co-morbidities were taken into consideration; e.g. pain duration correlated with standing HF power (r = −.217, p = 0.009). Age and BMI were negatively associated with standing HF power (r = −0.408, p < 0.0001 and r = −0.169, p = 0.017, respectively). In a subgroup analysis according to smoking status or LTPA among the patients, standing HR, HF power, SD1, or α_1_ did not differ from each other (p = ns for all).

## Discussion

We have shown that autonomic nervous system function assessed with the HRV method differs between sciatica patients referred to spine surgery and healthy controls. Vagally mediated HF power and SD1 were significantly lower in the patients than in the healthy controls, whereas the short-term fractal scaling exponent α_1_, indicating sympathovagal balance, was higher in the patients than in the controls after adjusting for potential confounders. Impaired functional capacity caused by pain or pain intensity was not associated with HRV among the patients, but HF power correlated negatively with LBP duration. Since altered HRV has been shown to have a prognostic value in both healthy subjects and various patient groups, the present results may provide important clues for understanding and considering the contribution of autonomic nervous system function in sciatica patients referred to spine surgery.

### Heart rate variability among low back patients

It is well documented that gender, BMI, smoking, and physical activity affects HRV [24-27]. Although there were more smokers, higher BMI, less high LTPA, and more men in the patient group than in the controls, HRV still differed between the groups when these potential confounders were accounted for. Additionally, the association remained evident after accounting for underlying co-morbidities and in separate sub-analysis where the patients with and without HNP-induced sciatica were compared. Taken together, obvious differences between the patient and control groups were observed in pain intensity (VAS score) and perceived disability (Oswestry), emphasizing the independent role of pain in HRV.

In a previous study, higher HF power was observed among patients with chronic LBP whose Oswestry score was less than 20% compared with patients whose score was 20–40%, but HF power did not differ between patients whose VAS score was 0–5 vs. 6–10 [10]. In the present study, we could not find an association between HF power and Oswestry or VAS score, whereas HF power correlated with pain duration, i.e. decreased vagal outflow was related to prolonged pain. Since in our study attenuated vagal activity during orthostatic stress was not linked to pain intensity or impaired functional capacity, it might be aggravated by the chronicity of the disease measured by the duration of pain. This finding may suggest that the dysfunction of the autonomic nervous system is a feature of the current pain condition and not related to pain severity *per se.*

### Potential mechanisms for decreased heart rate variability among the patients

It has been evidenced for a long time that various states of chronic pain, including LBP, are associated with signs of altered autonomic nervous system function, but the physiological mechanisms remain unclear. A recent study showed that HRV was reduced in complex regional pain syndrome (CRPS) patients compared with controls [8]. During orthostatic stress, patients with CRPS were not able to preserve cardiac output and they had an exaggerated increase in total peripheral resistance. Interestingly, the hemodynamic changes correlated with pain duration but not with pain intensity, e.g. a tilt-induced increase in total peripheral resistance correlated positively with pain duration [8]. Furthermore, in patients with chronic widespread pain (CWP), lower HRV is associated with higher pain intensity [28]. Interestingly, a recent study by Kang et al. showed that different disease profiles can be determined by clustering distinct features in patients with chronic neck pain [29]. According to this study, a high degree of disability associated with low HRV, high pain intensity, older age, poor sleep quality and high psychological distress. These findings may suggest a general autonomic imbalance in both CRPS, CWP and chronic neck pain patients, which may also be the status of our sciatica patients referred to spine surgery.

Recent studies have shown that the production of proinflammatory markers within the nucleus pulposus may be an important mediator in discogenic pain. Elevated levels of serum interleukin-6 (IL-6) and tumor necrosis factor-α (TNF-α) in patients with a herniated lumbar intervertebral disc [30] or elevated IL-6 in patients with lumbar radiculopathy [31], compared with healthy subjects, have been observed. Interestingly, autonomic dysregulation, expressed as depressed HRV, has been linked to increased IL-6 in healthy individuals as well as in those with a cardiovascular disease [32,33]. Therefore, it could be hypothesized that autonomic dysfunction observed in the sciatica patients in the present study may be related to increased cytokine production by the immune system. However, this should be confirmed in future studies.

### Methodological considerations

In the present study, we assessed HRV in a supine position and also during orthostatic stress in a standing position. It is well documented that HRV is susceptible to saturation when measured at a low HR [34,35], which is usually the case in a supine position, and it may therefore be unable to detect changes in cardiac vagal outflow [36]. Additionally, vagal and sympathetic regulation operates in a reciprocal fashion during a sympathetic orthostatic stimulus [37,38]. Therefore, a standing position might be a more favorable condition than supine for measuring autonomic function, since it could detect attenuated vagal outflow related to increased sympathetic activity, as well. Indeed, in the present study we found a clearer difference in HRV indices measured in a standing position then in a supine position.

It is also noteworthy that we measured the fractal scaling exponent α_1_ as an index of sympathovagal balance, because this index has been shown to provide information on autonomic interactions during various interventions and is less sensitive than spectral measures to trends and non-stationarities in HR behavior [23,39-41]. In the present study, the LF/HF ratio was higher in the patients than in the controls in a standing position, but after adjusting for sex, smoking, BMI, and LTPA, the difference between the groups disappeared (p = ns). However, the difference in α_1_ between the groups remained evident (p = 0.012) after adjusting for the above-mentioned covariates, indicating the role of both reduced vagal and increased sympathetic outflow among the patients.

### Limitations

Uncontrolled factors such as the night-time sleep or other psychological and physiological stressors may contribute to daily HRV. Our subjects were voluntary consecutive patients or healthy controls scheduled to daily hospital routine, and we were not able to control the above-mentioned factors. In addition, we could not control the use of pain medications, which may have an effect on HRV. Even though we may be able to evaluate pain medications according to hospital databases, we do not know the quality and amount of over-the-counter pain medication. However, we believe that most of the patients used pain medication at least to some extent while control population did not. This should “dilute” the results as pain medication is supposed to alleviate pain symptoms. Unfortunately, we do not have knowledge about use of physical therapy but we do not regard it an important confounder as it (if it works) has similar effect as pain medication. However, we analysed the data when underlying co-morbidities were excluded and the results were the same. Obviously, subjects with co-morbidities use more likely medication than subjects without co-morbidities. Therefore, we feel that, at least to some extent, medication is taken into consideration.

### Implications

Reduced HRV has been shown to be associated with the occurrence of various clinical events. Our results indicate that sciatica patients referred to spine surgery had autonomic dysregulation with decreased vagal activity and an increased sympathovagal balance compared with age-matched healthy controls. Therefore, it seems important to consider monitoring of the autonomic nervous system in addition to pain management in sciatica patients. We did not observe a significant relationship between impaired functional capacity caused by pain or pain intensity and HRV. In this respect, it can be suggested that HRV may reflect an independent intrinsic regulatory system of the autonomic nervous system that may be affected by the severity of the sciatic disease.

## Conclusions

These data suggest that sciatica patients referred to spine surgery have decreased HRV when compared with healthy subjects, and this may reflect a predisposition to impaired autonomic cardiovascular regulation.

## Abbreviations

BMI: Body mass index; CAD: Coronary artery disease; CRPS: Complex regional pain syndrome; CWP: Chronic widespread pain; HF: High frequency; HNP: Disc herniation induced nerve root impingement; HR: Heart rate; HRV: Heart rate variability; IL-6: Interleukin-6; LBP: Low back pain; LF: Low frequency; LF/HF: Low-to-high-frequency ratio; LTPA: Leisure-time physical activity; MRI: Magnetic resonance imaging; Oswestry: Low back pain disability index; SDNN: Standard deviation of all R-R intervals; SD1: Beat to beat R-R interval fluctuation; TNF-α: Tumor necrosis factor-α; VAS: Visual analog scale; α1=: Short-term fractal scaling exponent.

## Competing interests

The authors declare that they have no financial or non-financial competing interests.

## Authors’ contributions

JK, AJH and JS conceived the study and participated in its design. JK, AJH and JS had also full access to all of the data in the study and take responsibility for the integrity of the data and the accuracy of the data analyses and final version of the manuscript. JK, JP and EK coordinated and carried out the clinical data of the patients, participated the data analysis and drafted the manuscript. AMK and MPT participated in the analysis and interpretation of data and helped to draft the manuscript. AJH performed the statistical analysis together with AMK and MPT. All authors read and approved the final manuscript.

## Pre-publication history

The pre-publication history for this paper can be accessed here:

http://www.biomedcentral.com/1471-2474/14/149/prepub
